# Effect of preservation fluid contamination and associated possible donor-derived infections on early postoperative prognosis in kidney transplant recipients

**DOI:** 10.1186/s12866-024-03343-z

**Published:** 2024-05-30

**Authors:** Fei Zhang, Wenbo Wang, Jinbiao Zhong, Handong Ding, Guiyi Liao, Chaozhao Liang

**Affiliations:** 1 Department of Urology, the First Affiliated Hospital of Anhui Medical University, No. 218 Jixi Road, Shushan District, Hefei city, Anhui Province China; 2 Institute of Urology, the First Affiliated Hospital of Anhui Medical University, Hefei city, Anhui Province China; 3 Anhui Province Key Laboratory of Urological and Andrological Diseases Research and Medical Transformation, Anhui Medical University, Hefei city, Anhui Province China

**Keywords:** Preservation fluid, Possible donor-derived infections, Kidney transplant, Infections

## Abstract

**Background:**

The study aims to analyze the epidemiology of preservation fluid (PF) contamination and investigate the impact of PF contamination and possible donor-derived infections(p-DDI) on early postoperative prognosis in kidney transplant (KT) recipients.

**Methods:**

A total of 256 PF samples were collected for microbiological evaluation from all KT recipients who received deceased donor donations in our hospital from June 2018 to August 2022. Data on the baseline and clinical characteristics of these PF corresponding to recipients and donors were extracted from the electronic medical record. It mainly included the early postoperative complications and prognosis of KT recipients.

**Results:**

From June 2018 to August 2022, 597 kidney transplants were performed in our center, with 260 recipients receiving kidney transplantation from donation after citizens’ death. A total of 256 samples of PF were collected, of which 64.5% (165/256) were culture positive, and 24.6% (63/165) of the culture-positive PF were polymicrobial contamination. A total of 238 strains were isolated, of which coagulase-negative staphylococci (CoNS) had the highest proportion of 34.0% (81/238), followed by Klebsiella pneumoniae with 20.6% (49/238) and Escherichia coli with 8.8% (21/238). Recipients with culture-positive PF had a significantly higher incidence of postoperative infection (55.8% vs. 20.9%, *P* < 0.001) and DGF (38.2% vs. 24.2%, *P* = 0.023). In addition, the incidence of p-DDI was 12.9% (33/256). CRKP was the most common pathogen causing p-DDI. The recipients who developed p-DDI had a higher rate of graft loss (9.1% vs. 0.4%, *P* < 0.001), mortality (12.1% vs. 3.1%, *P* = 0.018), and longer postoperative hospital stay (30 days (19.5–73.5) vs. (22 days (18–32), *P* < 0.05) compared with recipients who did not develop p-DDI.

**Conclusions:**

Culture-positive PF is potentially significant for KT recipients, and p-DDI may increase the risk of poor prognosis for recipients. Prophylactic anti-infective treatment should be actively performed for highly virulent or multidrug-resistant (MDR) pathogens (especially Carbapenem-resistant Klebsiella pneumoniae, CRKP) in PF to avoid the occurrence of p-DDI.

**Supplementary Information:**

The online version contains supplementary material available at 10.1186/s12866-024-03343-z.

## Introduction

Infection is a major cause of morbidity and mortality after organ transplantation [1], and transplant recipients are more susceptible to postoperative infection due to underlying causative organ failure and the need for prolonged immunosuppressive therapy after transplantation. Most infections in kidney transplant (KT) recipients originate in the recipient, however, early post-transplant infections can also be caused by donor factors, including infections caused by PF contamination [2]. Preservation fluid (PF) contamination includes pathogens present in the donor organ (endogenous) or exogenous pathogens introduced by contamination during organ acquisition, trimming, or implantation [3]. In addition, due to its biochemical properties, organ PF can allow pathogens to survive and promote their growth with potential transmission to recipients, becoming a potential route of donor-derived infection [2, 4]. However, the clinical impact of PF contamination in KT recipients has not been well described.

The incidence of PF contamination in solid organ transplant (SOT) reported in different studies varies widely, ranging from ∼ 10% to as high as > 90%. Although the incidence of PF contamination is high, the incidence of infection caused by PF contamination, i.e., possible donor-derived infections (p-DDI), is low [5]. A review and meta-analysis [2] that included 17 studies found that the overall incidence of culture-positive PF in SOT recipients was 37%, the incidence of p-DDI among recipients with culture-positive PF was 4%, and the mortality among recipients who developed p-DDI was 35%, suggesting that although PF contamination is common, it causes a low incidence of p-DDI. Therefore, p-DDI may have an important impact on KT recipients. The most common organism in culture-positive PF is coagulase-negative staphylococci (CoNS), however, *Enterobacteriaceae* are more virulent, have a higher frequency of multidrug resistance, and are strongly associated with p-DDI in recent studies [2, 3, 6].

Routine microbiological analysis of PF may help to identify recipients at risk of developing infections in the early post-transplant period [7]. Therefore, the objectives of this study were to evaluate (i) the incidence and microbial distribution of PF contamination; (ii) the impact of PF contamination on the early postoperative prognosis of KT recipients; and (iii) the incidence of p-DDI and its impact on the early postoperative prognosis of KT recipients.

## Materials and methods

### Study design and patient sample

We retrospectively analyzed the clinical data of 256 KT recipients who received donations after citizens’ death and their corresponding 135 donors at the First Affiliated Hospital of Anhui Medical University from June 2018 to August 2022 and the microbiological culture results of 256 PF. Recipients who received KT from living donors or who did not undergo PF culture were excluded from the study. This study was approved by our institutional ethics review board and was conducted in accordance with the “Declaration of Helsinki guidelines”. However, due to the retrospective design of this study, informed consent was waived by the ethics committee.

### Clinical data collection

Clinical data of recipients were reviewed by collecting electronic medical records to assess differences in baseline and clinical characteristics between donors and recipients. Recipient variables included age, sex, body mass index, diabetes mellitus, etiology of kidney failure, type of dialysis, induction therapy, length of intensive care unit (ICU) stay after transplantation, length of hospitalization post-KT, length of surgery, cold ischemia time, acute rejection, delayed graft function (DGF), infection after transplantation, graft loss, and death. The follow-up time after transplantation was 3 months to assess the recipient’s early prognosis. Donor variables included age, sex, cause of death, donor type, length of ICU stay before donation, and warm ischemia time. In addition, the results of the culture of the PF were analyzed to understand the contamination of the PF.

### Definition

Culture-positive PF was defined as the growth of any microorganism in the PF culture. Our study used the criteria proposed by the Centers for Disease Control and Prevention to define and classify infections [8]. In particular, the following factors were considered: pneumonia (including ventilator-associated infections), surgical site infections (SSIs), bloodstream infections (BSIs) (including catheter-associated infections), and urinary tract infections (UTIs). Diagnosis culture for bacterial or fungal infection in the blood, sputum (or other respiratory secretions), urine, or abdominal drainage fluid were performed based on clinical suspicion. The combination of positive specimen cultures and clinical findings was used to define the occurrence of infection. The classification of utilized organs was as follows: Chinese category I (C-I), donation after brain death (DBD); Chinese category II (C-II), donation after cardiac death (DCD); China Category III (C-III), China donation after brain death plus cardiac death (DBCD) [9]. P-DDI was defined when the pathogen during the recipient’s infection was identical to the organism cultured in the PF and had the same susceptibility profile [10]. Multidrug resistance (MDR) was defined as acquired resistance to at least one agent in three or more antimicrobial categories [11]. DGF was defined as a decrease in daily serum creatinine of less than 10% from the previous day for 3 consecutive days in the first postoperative week or serum creatinine failing to decrease to 400 mmol/L in the first postoperative week [5].

### Preservation fluids and methods used to detect microorganisms

Graft PF consisted of hypertonic purine citrate solution (S400, Shanghai, China) for graft perfusion during organ procurement and preservation during graft transportation, and kidneys were preserved by static refrigeration. Prior to kidney implantation, two samples of PF (15 ml) were collected from the bag containing the kidney, and each sample was inoculated under sterile conditions into aerobic and anaerobic blood-culture flasks for identification and susceptibility testing if microorganisms grew during the culture. Bacterial species were cultured and identified using the VITEK-2 system (bioMérieux, Marcyl’Etoile, France). Minimum inhibitory concentrations (MICs) were interpreted according to breakpoints from the Clinical and Laboratory Standards Institute [12].

### Immunosuppressive therapy and infection prevention regimens

All enrolled recipients received triple immunosuppression (tacrolimus or cyclosporine A, prednisone, and mycophenolate), with some recipients adding anti-thymocyte immunoglobulin induction. In addition, the recipients were given meropenem 1 g via an intravenous drip during the operation to prevent infection. Furthermore, cefoperazone sulbactam sodium was given postoperatively for preventive anti-infection for at least 7 days, along with antifungal drugs for preventive application for 2 weeks. Antimicrobial therapy was adjusted postoperatively according to the spectrum of resistance of microorganisms identified in recipient specimens and PF. The treatment was subsequently stopped in the absence of clinical signs of infection and microbial isolation in recipients.

### Statistical analysis

Statistical analysis was performed using SPSS software [Version 25.0; SPSS Inc, Chicago, IL, USA]. The means and standard deviations of quantitative variables were reported, as were the medians and interquartile ranges (IQRs). Independent sample t-test or Mann–Whitney U-test was used to compare quantitative variables between groups. Categorical variables are presented as frequencies and percentages. The chi-square test or Fisher’s exact test was used to compare categorical variables between groups, as appropriate. For all tests, *P* < 0.05 was considered statistically significant.

## Result

According to the medical records, 597 KT were performed between June 2018 and August 2022. In total, 256 recipients of deceased organ donations were included in the study, with 135 corresponding donors. There were 33 cases of p-DDI infection in 165 cases of culture-positive PF.

### Incidence and microbial distribution of culture-positive PF

The distribution of microorganisms isolated in PF is shown in Table 1. Among 256 PF, 165 were culture positive. The Incidence of culture-positive PF was 64.5% (165/256), among which 102 contaminated samples were monomicrobial and 63 were polymicrobial. A total of 238 strains were isolated, including 45.8% (109/238) Gram-positive coccus, 43.3% (103/238) Gram-negative bacilli, and 10.9% (26/238) fungus, with the highest proportion of CoNS among all pathogens, accounting for 34.0% (81/238), followed by 20.6% (49/238) *Klebsiella pneumoniae*, 8.8% (21/238) *Escherichia coli*, and 6.3% (15/238) *Enterococcus faecium*. *Candida albicans* was the most common fungus, accounting for 2.9% (7/238). MDR positive rate was high, accounting for 26.2% (67/256) of the isolates, of which *Klebsiella pneumoniae* was the most common, accounting for 58.2% (39/67), and 89.8% (35/39) were CRKP.

**Table 1:** Microorganisms isolated from preservation fluid samples

Microorganisms	No. Percent (%)
**Negative**	91 (35.5)
**Positive**	165 (64.5)
Monomicrobial	102 (61.8)
Polymicrobial	63 (38.2)
**Gram-positive coccus**	
*CoNS*	81 (34.0)
*Staphylococcus aureus*	4 (1.7)
*Enterococcus faecium*	15 (6.3)
*Enterococcus faecalis*	7 (2.9)
Others	2 (0.9)
**Gram-negative bacilli**	
*Klebsiella pneumoniae*	49 (20.6)
*Escherichia coli*	21 (8.8)
*Acinetobacter baumannii*	10 (4.2)
*Enterobacter cloacae*	7 (2.9)
*Pseudomonas aeruginosa*	3 (1.3)
Others	13 (5.5)
**Fungus**	
*Candida albicans*	7 (2.9)
*Candida parapsilosis*	6 (2.5)
*Candida tropicalis*	4 (1.7)
*Candida glabrata*	5 (2.1)
Others	4 (1.7)

Abbreviations: CoNS: coagulase negative staphylococcus

### Donor characteristics

The characteristics of donors are shown in Table 2, and the most common cause of death in both groups of donors was Brain trauma, and the most common type of organ donation was DBCD. The mean age of donors was similar between the two groups, 46.22 ± 11.82 years in the culture-positive PF donors and 50.00 ± 11.30 years in the culture-negative PF donors (*P* = 0.067). The length of ICU stay (median, 15 days; IQR, 9–18) was significantly longer in culture-positive PF donors than in culture-negative PF donors (median, 8 days; IQR, 6–10) (*P* < 0.05). The other variables were not significantly different between the two groups (*P* > 0.05).

**Table 2:** Comparison of baseline and clinical characteristics between culture-positive and culture-negative PF donors

Characteristics	Culture negative PF	Culture positive PF	*P* Value
	*N* = 50(%)	*N* = 85(%)	
Sex, male *n* (%)	22(44.0)	45(52.9)	0.316
Age(years)	46.22 ± 11.82	50.00 ± 11.30	0.067
**Cause of death, n (%)**			
Brain trauma	25 (50.0)	31(36.5)	0.123
Cerebrovascular accidents	11(22.0)	28(32.9)	0.176
Brain tumor	7(14.0)	13(15.3)	0.838
Others	7(14.0)	13(15.3)	0.838
**Donor type, n (%)**			
DBD	8(16.0)	21(24.7)	0.234
DCD	17(34.0)	20(23.5)	0.188
DBCD	25(50.0)	44(51.8)	0.843
Length of ICU stay (days)	8(6–10)	15(9–18)	***P*** **<0.001**
Warm ischemia time(min)	9(8–11)	9(8–10)	0.120

Abbreviations: PF: preservation fluid; DBD: donation after brain death; DCD: donation after cardiac death; DBCD: donation after brain death plus cardiac death

### Effect of PF contamination on KT recipients

Within three months after kidney transplantation, 111 of 256 recipients developed an infection at a rate of 43.4% (111/256). There were no significant differences in baseline characteristics such as age, sex, BMI, diabetes mellitus, and type of dialysis between recipients of culture-positive PF and culture-negative PF (Table 3). The postoperative infection rate of culture-positive PF recipients was significantly higher than that of culture-negative PF recipients (55.8% vs. 20.9%, *P* < 0.001). However, when different infection events were involved, the incidence of culture-positive PF recipients’ SSIs was significantly higher than that of negative result recipients (31.5% vs. 6.6%, *P* < 0.001). Similar results were observed in the incidence of UTIs between the two groups (29.7% vs. 14.3%, *P* = 0.006). There was no significant difference in the incidence of Pneumonia and BSIs between the two groups. Culture-positive PF recipients had a higher incidence of DGF than negative outcome recipients (38.2% vs. 24.2%, *P* = 0.023). In contrast, there was no significant difference between the two groups in terms of mortality, the incidence of acute rejection, and graft loss.

**Table 3:** Comparison of baseline and clinical characteristics between culture-positive and culture-negative PF recipients

Characteristics	Culture negative PF	Culture positive PF	*P* Value
	*N* = 91(%)	*N* = 165(%)	
Sex, male	62(68.1)	120(72.7)	0.438
Age(years)	41.42 ± 11.10	39.76 ± 10.11	0.227
BMI (kg/m2)	21.80 ± 2.95	21.68 ± 3.57	0.776
Diabetes mellitus	10(11.0)	24(14.5)	0.422
**Aetiology of kidney failure**			
HTA	10(11.0)	12(7.3)	0.310
DM	2(2.2)	11(6.7)	0.119
Glomerulonephritis	54(59.3)	108(65.4)	0.331
Others	25(27.5)	34(20.6)	0.212
**Type of dialysis**			
HD	71(78.0)	128(77.6)	0.935
PD	20(22.0)	37(22.4)	0.935
ATG induction	51(56.0)	75(45.4)	0.105
Length of ICU stay (days)	0(0–1)	0(0–1)	0.064
Length of hospitalization post-KT (days)	23(20–32)	22(18–37)	0.363
Length of surgery (h)	4(3.5-5.0)	4(3.5-5.0)	0.934
Cold ischemia time (h)	9.7(8.0–12.0)	9(8.0-12.5)	0.894
Overall infections	19(20.9)	92(55.8)	***P*** **<0.001**
Pneumonia	7(7.7)	17(10.3)	0.493
BSIs	9(9.9)	30(18.2)	0.077
UTIs	13(14.3)	49(29.7)	**0.006**
SSIs	6(6.6)	52(31.5)	***P*** **<0.001**
Acute rejection	41(45.1)	65(39.4)	0.379
DGF	22(24.2)	63(38.2)	**0.023**
Graft loss	0(0)	4(2.4)	0.134
Death	1(1.1)	10(6.1)	0.061

Abbreviations: PF: preservation fluid; BMI: body mass index HTA: hypertension; DM: diabetes mellitus; HD: hemodialysis; PD: peritoneal dialysis; ATG: anti-thymocyte globulin; BSIs: bloodstream infections; UTIs : urinary tract infections; SSIs : surgical site infections; DGF: delayed graft function

### Effect of p-DDI on KT recipients

Pathogens identical to microorganisms cultured in the PF and with the same drug-sensitive profile were isolated from postoperative specimen cultures from 33 postoperatively infected recipients, with a p-DDI incidence of 12.9% (33/256). The time to first positive culture was 4 (IQR: 2-7.5) days after transplantation in p-DDI infected recipients and 10 days (IQR: 6.75-16) in other infected recipients except p-DDI infected recipients. One recipient had a multi-bacterial transmitted infection (CRKP and *Enterococcus faecium*), and the remaining 32 had a mono-bacterial transmitted infection. A total of 34 pathogens developed p-DDI, including CRKP (*n* = 20), *Enterococcus faecium* (*n* = 5), *Acinetobacter baumannii* (*n* = 1), *Candida tropicalis* (*n* = 4), *Candida krusei* (*n* = 2), *Candida albicans* (*n* = 1), and *Candida glabrata* (*n* = 1), of which all bacteria were MDR. There were no significant differences between recipients with and without p-DDI in terms of baseline characteristics such as age, sex, and type of dialysis. The specific characteristics of the two group recipients are shown in Table 4. The length of hospital stay after kidney transplantation was significantly longer in recipients with p-DDI (median, 30 days; IQR, 19.5–73.5) than in recipients without p-DDI (median, 22 days; IQR, 18–32) (*P* < 0.05). The graft loss rate and mortality of recipients with p-DDI were significantly higher than those without p-DDI (9.1% vs. 0.4%, *P* < 0.001; 12.1% vs. 3.1%, *P* = 0.018). Among recipients who developed p-DDI, three recipients experienced kidney allograft loss but survived and four recipients died, all of whom were associated with CRKP-transmitted infections. Another recipient who developed *Candida albicans*-transmitted infections developed bleeding at the anastomotic site of the transplanted kidney. After undergoing surgical treatment for anastomotic reconstruction and receiving aggressive antifungal therapy, the recipient was discharged successfully.

**Table 4:** Comparison of baseline and clinical characteristics between recipients with p-DDI and those without p-DDI

Characteristics	Recipients with *p*-DDI	Recipients without *p*-DDI	*P* Value
	*N* = 33(%)	*N* = 223(%)	
Sex, male	24(72.7)	158(70.8)	0.824
Age(years)	39.70 ± 10.37	40.45 ± 10.51	0.701
BMI (kg/m2)	22.27 ± 2.79	21.64 ± 3.43	0.319
Diabetes mellitus	6(18.2)	28(12.6)	0.374
**Aetiology of kidney failure**			
HTA	2(6.0)	20(8.9)	0.578
DM	3(9.1)	10(4.5)	0.261
Glomerulonephritis	22(66.7)	140(62.8)	0.666
Others	6(18.2)	53(23.8)	0.477
**Type of dialysis**			
HD	22(66.7)	177(79.4)	0.102
PD	11(33.3)	46(20.6)	0.102
ATG induction	12(36.4)	114(51.1)	0.114
Length of surgery (h)	4(4–5)	4(3.5-5.0)	0.208
Cold ischemia time (h)	10(8.5–13)	9(8–12)	0.187
Acute rejection	14(42.4)	92(41.3)	0.899
DGF	15(39.4)	70(31.4)	0.109
Graft loss	3(9.1)	1(0.4)	***P*** **<0.001**
Death	4(12.1)	7(3.1)	**0.018**
Length of ICU stay (days)	0(0–1)	0(0–1)	0.617
Length of hospitalization post-KT (days)	30(19.5–73.5)	22(18–32)	**0.021**

Abbreviations: p-DDI: possible donor-derived infections; BMI: body mass index HTA: hypertension; DM: diabetes mellitus; HD: hemodialysis; PD: peritoneal dialysis; ATG: anti-thymocyte globulin; DGF: delayed graft function

## Discussion

The incidence of donor kidney PF contamination varies widely among transplant centers where PF is cultured, ranging from 7.2 to 77.8% [5, 13, 14]. The routine culture of PF can be considered a tool to provide information on graft contamination. In our study, the PF contamination incidence was 64.5% (165/256), with CoNS and *Enterobacteriaceae* being the most common pathogens in PF cultures, which is consistent with the results of previous studies [13, 15, 16]. Although the incidence of PF contamination was high, the incidence of p-DDI was low at 12.8% (33/256), and the rate of graft loss and mortality of recipients was high when p-DDI occurred. The pathogen that develops p-DDI is predominantly CRKP. To our knowledge, this study reports data on the largest kidney transplant recipient of p-DDI caused by CRKP to date.

The incidence of PF contamination in this study was 64.5%, which was similar to the results of previous studies [5, 13, 14]. Yu et al. [5] evaluated 1002 PF samples from 517 kidney donors for contamination in a retrospective study and found a 77.8% incidence of PF contamination in the study. In a multicenter prospective cohort study conducted by Oriol et al., the culture of PF from 622 recipients undergoing solid organ transplantation was found to have a positive culture rate of 62.5% [15]. The incidence of contamination of PF in SOT recipients reported in different studies varies, the reason for this significant difference in contamination rates may be derived from different transplantation procedures, sample sizes, study design types (higher contamination rates in prospective studies) [2], types of PF used during transplantation, whether antibiotics [7] are added to the PF, timing of culture collection (during organ acquisition, trimming of organs, and before implantation), and detection methods [15] for related pathogens. Some studies have investigated the risk factors of PF contamination in order to reduce the incidence of PF contamination. Corbel et al. [17] analyzed the culture results of PF of 4487 KT recipients in France, in multivariate analysis, it was found that for KT from donation after citizens’ death, factors associated with increased risk of PF contamination included intestinal perforation of the donor during procurement, multiorgan procurement, and en bloc transplantation. Using a perfusion pump and donor antibiotic treatment lowers the risk of PF contamination. This study found that culture-positive PF donors had significantly longer ICU stays than culture-negative PF donors, which is consistent with the findings of Cerutti et al., who found that prolonged ICU stays increase the risk of donor infection and thus increase the chance of PF contamination [18]. The aforementioned data shows that the donor’s condition significantly impacts the contamination of the PF. More effective donor management and timely organ donation are beneficial in reducing the incidence of PF contamination.

CoNS was this study’s most common pathogen in PF cultures, consistent with previous findings [15, 19]. Reticher et al. [19] analyzed the culture results of 152 kidney allograft PF, they found that 80% of the positive microorganisms were consistent with the skin flora, of which CoNS was the most common, accounting for 56% of all microbial growth. The higher detection rate of CoNS indicates that contamination in the PF is likely to be derived from exogenous pathogens and less likely from the donor organ itself. The proportion of MDR in PF culture results has increased in recent years. Li et al. [14] collected 864 graft kidney PF for culture, and MDR-Gram-negative bacilli were cultured in 80 of them, the most common being *Klebsiella pneumoniae*, which was consistent with our results. The emergence of *Enterobacteriaceae* in the PF likely suggests that the donor developed an infection during organ procurement [6]. Fungus accounts for a relatively small proportion of culture-positive microorganisms in PF compared to bacteria. Moreover, the proportion of fungus in this study was 10.9% (26/238), consistent with previous studies reporting 6.4-14.4%, with *Candida albicans* being the most common fungus [5, 6].

Our study found that culture-positive PF recipients had a higher incidence of postoperative infection than culture-negative PF recipients (55.8% vs. 20.9%, *P* < 0.001), with no significant differences between the two groups in terms of incidence of graft loss and mortality. A retrospective analysis of 331 SOT recipients was performed by Yansouni et al. [20], they found that postoperative infection was more common in culture-positive PF SOT recipients regardless of transplant type, which is consistent with our findings. Similarly, differences in graft loss rates and mortality between culture-positive PF KT recipients and negative KT recipients were not observed in this study. In a prospective multicenter cohort study from Spain, Oriol et al. [21] showed that culture-positive PF recipients had a higher 90-day cumulative infection rate, acute rejection rate, and mortality than negative recipients. However, our study found no difference in acute rejection rate or mortality between the two groups. Some studies also found no correlation between the contamination of PF and the increased risk of postoperative recipient infection [7, 19], which may be related to the postoperative follow-up time and sample size. Contamination of PF was also reported to be associated with impaired graft function [7, 22]. A higher incidence of DGF was also found in culture-positive PF recipients compared with culture-negative recipients in our study (38.2% vs. 24.2%, *P* = 0.023).

Regarding infection transmission, 33 recipient postoperative specimen culture results were consistent with donor kidney PF culture results. Considering p-DDI, the incidence of p-DDI was 12.9% (33/256), which was consistent with the incidence of p-DDI reported in the previous literature from 2.9–17.8% [5, 6]. The mortality of 12.1% (4/33) of p-DDI recipients in this study was lower than that of 35 – 50%, as reported in previous studies [2, 6, 14], this could be due to the timely use of antibiotics for the prophylactic treatment of pathogens cultured in the PF in our center. We found that recipients with p-DDI had higher graft loss rates and mortality and longer postoperative hospital stay than those without p-DDI. Janny et al. [6] analyzed data from 477 liver transplant recipients and found higher mortality in recipients who developed p-DDI than those without p-DDI, however, no significant difference was found in the postoperative length of stay. Previous studies have found highly virulent ESKAPE pathogens (*Enterococcus faecalis*, *Staphylococcus aureus*, *Klebsiella pneumoniae*, *Acinetobacter baumannii*, *Pseudomonas aeruginosa*, and *Enterobacteriaceae*) in PF can significantly increase the incidence of p-DDI [5, 23]. Most pathogens that developed p-DDI in this study were CRKP, followed by *Enterococcus faecium*, consistent with previous findings [24]. The proportion of MDR in these pathogens was very high, it may evade perioperative antibiotics to donors and recipients and lead to an increased risk of p-DDI, low-virulence CoNS was not found to be associated with p-DDI [7, 13, 19]. Fungal development of p-DDI has previously been reported to cause severe effects on recipients, including fungal arteritis or anastomotic aneurysms, resulting in vascular rupture, graft loss, and even recipient death [25, 26]. Eric et al. [25] collected more than 2,000 PF and found that 8 of 28 recipients with positive fungal cultures in PF developed p-DDI, including 6 with hepatic aneurysms and 2 with Candida peritonitis, with a one-year postoperative mortality rate of 62.5% (5/8). Fungal p-DDI occurred in 8 recipients in our study, the kidneys of these 8 recipients were derived from 4 corresponding donors who were positive for PF fungal culture, and the culture results corresponded to the pathogens of these 8 recipients who developed p-DDI infection, respectively. Only one case resulted in an anastomotic hemorrhage and the graft survived after active treatment, which may be attributed to the prophylactic antifungal treatment to the recipients for 2 weeks postoperatively.

The high incidence of positive PF cultures and the low incidence of p-DDI are reasons why some authors do not recommend routine PF culture, and they believe that the benefit of treatment is very low, with increased patient expenditure and risk of resistant microorganisms [16, 27]. However, mortality from PF-related infections reported in other studies recommends PF culture in transplant centers along with short-term prophylactic antibiotic therapy in SOT recipients with positive PF culture [6, 28]. At present, there are no international clinical guidelines for the evaluation of PF or the use of prophylactic antibiotics. A national questionnaire study was conducted in France on whether to prescribe antibiotics for prophylaxis when bacteria were positive in PF culture, the results showed whether to prescribe antibiotics varied greatly among respondents, largely depending on personal clinical experience [29]. Oriol et al. [21] conducted a prospective multicenter cohort study of 622 SOT recipients in which positive organisms cultured in PF were classified as high and low risk, they discovered that prophylactic antibiotic therapy only improved the outcome of recipient infection, graft loss, and acute rejection when PF was cultured as a high-risk microorganism, in addition, it did not increase the proportion of ESBL isolates and MDR strains in subsequent infections in these SOT recipients. In another study analyzing the difference in the risk of p-DDI between SOT recipients receiving and not receiving prophylactic anti-infective therapy, the pathogenic bacteria culture-positive PF was 2.0%, and saprophytic bacteria culture-positive PF was 0% [2]. However, the study conducted by Ranghino et al. found that prophylactic antibiotic treatment based on culture results of PF did not reduce the incidence of p-DDI [30]. In cases where cultures of PF are positive for fungus, several studies have recommended systemic prophylactic antifungal therapy because of the risk of fungal arteritis and aneurysms [25, 29].

This study has several limitations. First, it is a single-center retrospective study. Second, because the cause of PF contamination cannot be determined, it is difficult to fully identify the relationship between PF contamination and postoperative infection and can only demonstrate its potential association. Third, information on donor microbiological cultures and antibiotic use was not available. Finally, genotyping technology is required to obtain objective evidence of DDI, unfortunately, this technology is not widely available at our hospital, so we used the definition of p-DDI, which may lead to overdiagnosis of potential donor-derived infections.

## Conclusion

In summary, the routine culture of organ PF can provide information about the contamination of transplanted organs, whether transmitted by the donor or secondary to transplant surgery. Therefore, we recommend routine culture of PF to ensure that potentially essential pathogens are not missed. For KT recipients with PF culture results of highly virulent or MDR pathogens (especially CRKP), preventive anti-infective treatment should be actively performed according to drug susceptibility results to avoid the occurrence of p-DDI. Since most of the pathogens that cause p-DDI are CRKP, we recommend that the inpatient departments of the donors should take active measures to treat and prevent donor CRKP infection before donation, so as to improve the early prognosis of kidney transplant recipients. In addition, prospective multicenter studies of PF contamination, including molecular epidemiology, are needed to determine the best strategies to prevent and manage subsequent infections.

### Electronic supplementary material

Below is the link to the electronic supplementary material.

Supplementary Material 1

## Data Availability

The datasets used and/or analysed during the current study are available from the corresponding author on reasonable request.

## Abbreviations

BSIs: Bloodstream infections
CoNS: Coagulase-negative staphylococci
CRKP: Carbapenem-resistant Klebsiella pneumoniae
DDI: Donor-derived infections
DCD: Donation after cardiac death
DBD: Donation after brain death
DBCD: Donation after brain death plus cardiac death
DGF: Delayed graft function
MDR: Multidrug-resistant
SOT: Solid organ transplant
SSIs: Surgical site infections
KT: Kidney transplant
PF: Preservation fluid
p-DDI: Possible donor-derived infections
UTIs: Urinary tract infections
ICU: Intensive care unit
