# Supplementary material for: Effect of preservation fluid contamination and associated possible donor-derived infections on early postoperative prognosis in kidney transplant recipients
Source: BMC Microbiol. 2024 May 30;24:189. doi: 10.1186/s12866-024-03343-z (PMC11137905; doi:10.1186/s12866-024-03343-z)
Supplement: Supplementary file 1 — Supplementary Material 1 [file 12866_2024_3343_MOESM1_ESM.docx]

**Table S1. Infections site and outcomes for 26 recipients from the corresponding 13 donors.**

| **Recipients** | **Infection sites** | **DGF** | **Outcomes** |
| --- | --- | --- | --- |
| 1 | BSIs | DGF | Death |
| 2 | Pneumonia, UTIs, SSIs | DGF | Survival |
| 3 | Pneumonia, UTIs, SSIs | DGF | Death |
| 4 | Pneumonia, UTIs, SSIs | / | Survival |
| 5 | BSIs, UTIs, SSIs | / | Death |
| 6 | UTIs, SSIs | DGF | Survival |
| 7 | UTIs, SSIs | DGF | Survival |
| 8 | BSIs | DGF | Graft loss |
| 9 | SSIs | DGF | Survival |
| 10 | BSIs, SSIs | / | Death |
| 11 | Pneumonia, UTIs, SSIs | DGF | Survival |
| 12 | UTIs, SSIs | / | Survival |
| 13 | BSIs, UTIs, SSIs | DGF | Graft loss |
| 14 | SSIs | / | Survival |
| 15 | Pneumonia, BSIs, SSIs | DGF | Graft loss |
| 16 | SSIs | DGF | Survival |
| 17 | SSIs | DGF | Survival |
| 18 | BSIs, UTIs, SSIs | DGF | Survival |
| 19 | SSIs | / | Survival |
| 20 | BSIs | DGF | Survival |
| 21 | SSIs | / | Survival |
| 22 | UTIs, SSIs | / | Survival |
| 23 | BSIs, SSIs | / | Survival |
| 24 | BSIs, SSIs | / | Survival |
| 25 | BSIs, UTIs, SSIs | DGF | Survival |
| 26 | SSIs | / | Survival |

**Abbreviations:** bloodstream infections, BSIs; surgical site infections, SSIs; urinary tract infections, UTIs; delayed graft function, DGF. **Note:** Recipients 1-26 corresponds to Donors 1-13, respectively. “/” represents non-DGF.
